# Accelerating discovery: A novel flow cytometric method for detecting fibrin(ogen) amyloid microclots using long COVID as a model

**DOI:** 10.1016/j.heliyon.2023.e19605

**Published:** 2023-08-29

**Authors:** Simone Turner, Gert Jacobus Laubscher, M Asad Khan, Douglas B. Kell, Etheresia Pretorius

**Affiliations:** a Department of Physiological Sciences, Faculty of Science, Stellenbosch University, Stellenbosch, Private Bag X1, Matieland, 7602, South Africa; b Mediclinic Stellenbosch, Stellenbosch, 7600, South Africa; c Respiratory Medicine, Manchester University Hospitals, Wythenshawe Hospital, Manchester, M23 9LT, United Kingdom; d Department of Biochemistry and Systems Biology, Institute of Systems, Molecular and Integrative Biology, Faculty of Health and Life Sciences, University of Liverpool, L69 7ZB, UK; e The Novo Nordisk Foundation Centre for Biosustainability, Technical University of Denmark, Kemitorvet 200, 2800, Kgs Lyngby, Denmark

**Keywords:** Long COVID, Amyloid microclots, Imaging flow cytometry, Fluorescence

## Abstract

Long COVID has become a significant global health and economic burden, yet there are currently no established methods or diagnostic tools to identify which patients might benefit from specific treatments. One of the major pathophysiological factors contributing to Long COVID is the presence of hypercoagulability; this results in insoluble amyloid microclots that are resistant to fibrinolysis. Our previous research using fluorescence microscopy has demonstrated a significant amyloid microclot load in Long COVID patients. However, this approach lacked the elements of statistical robustness, objectivity, and rapid throughput. In the current study, we have used imaging flow cytometry for the first time to show a significantly increased concentration and size of these microclots. We identified notable variations in size and fluorescence between microclots in Long COVID and those of controls even using a 20× objective. By combining cell imaging and the high-event-rate and full-sample analysis nature of a conventional flow cytometer, imaging flow cytometry can eliminate erroneous results and increase accuracy in gating and analysis beyond what pure quantitative measurements from conventional flow cytometry can provide. Although imaging flow cytometry was used in our study, our results suggest that the signals indicating the presence of microclots should be easily detectable using a conventional flow cytometer. Flow cytometry is a more widely available technique than fluorescence microscopy and has been used in pathology laboratories for decades, rendering it a potentially more suitable and accessible method for detecting microclots in individuals suffering from Long COVID or conditions with similar pathology, such as myalgic encephalomyelitis.

## Highlights

• Imaging flow cytometry utilizing a novel rapid flow cytometric detection method is a promising method for detecting amyloid fibrin(ogen) microclots in conditions associated with clotting pathology.
• While this investigation represents a preliminary study, it lays the foundation for further research into the method's utility as a diagnostic tool for individuals with Long COVID.
• Though challenging, future research can explore potential correlations between microclot detection and specific Long COVID symptoms and comorbidities, enhancing our understanding of the pathophysiology and tools to detect this condition.
• The method's ability to evaluate amyloid microclot presence, concentration, and size offers valuable insights for clinical applications.

## 1 Introduction

Long COVID has become a major global health and economic burden [[1], [2], [3], [4], [5], [6], [7], [8], [9], [10]]. A major contributor to the exponential rise in numbers is arguably the absence of a clear suite of detection methods and diagnostic tools to identify patients that may benefit from specific treatments. The causes of the persistent symptoms of Long COVID include immune dysregulation, autoantibodies, immune dysregulation, reactivation of latent viruses, viral persistence, organ damage, and hypercoagulability [8,11,12].

The presence of clotting pathology in the form of fibrinaloid microclots, hyperactivated platelets and endothelial dysfunction represents a key target for both the diagnosis and the treatment of the condition [1,8,11,12]. The spike protein from the virus has a key role in causing the various pathologies; it can activate clotting factors [13], it is itself amyloidogenic [14], and it may have direct protein-protein interactions with the main plasma clotting protein, fibrinogen [2,15]. These plasma protein interactions, which can also be driven by bacterial cell wall components [16,17], result in the formation of amyloid fibrin microclots (fibrinaloids) that are unusually resistant to fibrinolysis. Receptors on platelets and endothelial cells may also interact with viral inflammagens and circulating inflammatory molecules [15,[18], [19], [20], [21], [22]]. These interactions may lead to widespread platelet hyperactivation and endothelial damage. Pathological platelets may also form platelet complexes with various immune cells. Hence there are potentially several ways by which platelets, endothelial cells and plasma proteins may trigger an immune-thrombotic cascade [23].

Previously, we provided fluorescence microscopic evidence that there is a significant insoluble fibrin amyloid microclot load along with platelet hyperactivation in both acute and Long COVID [[1], [2], [3], [4], [5], [6],^15^,^21^,[24], [25], [26], [27]]. We also proposed a clotting and grading system for use in the absence of a general quantitative pathology laboratory method [25]. We also previously confirmed entrapped inflammatory molecules within these samples using proteomics analysis [4,6].

Whilst fluorescence microscopy can identify microclots and platelet hyperactivation, it lacks statistical robustness, objectivity, and high throughput. Being a research tool, it is not readily accessible to patients or clinicians. However, flow cytometric methods are widely available, and since the 1980s have been capable of detecting micron-sized objects [28]. Flow cytometry is a powerful diagnostic tool that has broad applications in various fields including immunology, molecular biology, bacteriology, virology, cancer biology, and infectious disease monitoring [29]. Here we describe an imaging flow cytometric method that we have developed to measure the burden of fibrinaloid microclots in platelet-poor plasma (PPP).

Whilst we used an imaging flow cytometer, the results indicate that these signals will be readily detectable on a non-imaging instrument. We provide evidence that supports the use of imaging flow cytometry as a novel method for microclot detection, with the potential to serve as a diagnostic tool. However, further research is required to establish its clinical utility.

## 2 Materials and methods

### 2.1 Ethical clearance

Ethical clearance for the study was obtained from the Health Research Ethics Committee (HREC) of Stellenbosch University (South Africa) (references: B21/03/001_COVID-19, project ID: 21911 (Long COVID registry) and N19/03/043, project ID 9521 with yearly re-approval). Participants were either recruited for a blood donation via the Long COVID registry or identified from our clinical collaborator's practice. Except for 2 control samples, all other samples were stored samples that were initially collected for our proteomics analysis [4]. The link to the Long COVID registry is at https://airtable.com/shrtucasatrINAL7K. The online registry features dropdown boxes offering a range of selection options. For instance, there are “yes” or “no” options, and selecting “yes” prompts a request for additional information. Additionally, participants have the option to provide a blood sample, should they decide to do so. Please refer to the supplementary material for further details. The experimental objectives, risks, and details were explained to volunteers and informed consent was obtained prior to blood collection. Strict compliance to ethical guidelines and the principles of the Declaration of Helsinki, South African Guidelines for Good Clinical Practice, and Medical Research Council Ethical Guidelines for Research were kept for the duration of the study and for all research protocols.

### 2.2 Patient demographics

#### 2.2.1 Participant identification for flow cytometry analysis

For flow cytometry analysis, we randomly selected stored blood samples from Long COVID and control patients; from these samples, we obtained platelet poor plasma (PPP). We included blood from 20 healthy individuals to serve as controls (7 males; 13 females; mean age 46 (±11)) and 40 Long COVID patients (18 males; 22 females; mean age 48 (±14)). Healthy participants did not smoke, did not suffer from coagulopathies, and were not pregnant. The questionnaire collected information regarding their age, gender, pre-existing comorbid conditions, regular medication, information regarding the onset, diagnosis, and severity of their acute COVID-19 illness, and their self-reported Long COVID symptoms. Vaccination status was also noted. Long COVID patients were identified based on self-reported symptoms which developed after acute infection.

#### 2.2.2 Blood sample collection and sample preparation

Either a qualified phlebotomist or a medical practitioner drew blood samples. To obtain PPP, whole blood (WB) from sodium citrate tubes was centrifuged at 3000×*g* for 15 min at room temperature, and the supernatant PPP samples were collected in 1.5 mL Eppendorf tubes and stored at −80 °C. On the day of analysis, the stored PPP was brought to room temperature and exposed to thioflavin T (ThT), a fluorescent dye that binds to amyloid protein, at a final concentration of 0.03 mM (obtained from Sigma-Aldrich, St. Louis, MO, USA). The exposure lasted 30 min, during which time the plasma was protected from light.

#### 2.2.3 Flow cytometry

For data acquisition, we used an Amnis® FlowSight® Imaging Flow Cytometer from Luminex. Imaging flow cytometry combines the advantages of microscopy's single-cell image acquisition with the high-event-rate capacity, quantification, and statistical reliability of traditional flow cytometry. For acquisition, a template was created where the 405 nm and 488 nm lasers were turned on and a gate was established to collect all ThT positive (ThT +) events by using a negative control (water and ThT), a second negative control (PPP without ThT), and a standard PPP control as reference **(see supplementary material)**. To reduce background noise and file size, we only collected events within the ThT-positive gate for analysis. The brightfield images were viewed in Channel 1 and ThT-positive events were viewed in Channel 7 using the 405 laser. All samples were acquired using the same acquisition template for 5 min each. Given that the number of microclots per mL ranged from 21,000–52,000 (Table 2) per sample, this typically involved the detection of 400–3000 microclots per sample. The time required for IDEAS to analyse a sample using the analysis template was approximately 2 min, which could vary based on the number of events in the sample. Overall, the total time required to process a single sample from acquisition to analysis was approximately 7 min.

**Table 1:** Sample demographics and considerations.

Previously stored platelet poor plasma	
Mean age [and standard deviation (SD)] of controls and Long COVID patients	
Controls (n = 20)	47 (±11)
Long COVID (n = 40)	48 (±14)
**Gender of controls and Long COVID patients**	
Controls (n = 20)	7 males; 13 females
Long COVID (n = 40)	18 males; 22 females
**Vaccination status of controls and Long COVID patients before blood collection**	
Percentage of controls vaccinated (n = 20)	65% vaccinated
Percentage of Long COVID patients vaccinated (n = 40)	Out of the total of 40 individuals, vaccination status was unknown for 2, while 74% (28 out of 38) had been vaccinated.
**Symptoms of Long COVID patients (n = 40)**	
**Symptom**	**% In 40 Long COVID patients**
Fatigue	72%
Brain fog/forgetfulness/poor concentration	64%
Dyspnoea	52%
Arthralgia/Myalgia	48%
Depression/Anxiety	40%
Sleep disturbance	24%
Anosmia	24%
Dysgeusia	24%
Arrythmias/Palpitations	24%
Recurring Chest Pain	24%
Digestive problems	16%
Low oxygen levels	12%
Kidney problems	8%
**Co-morbidities of Long COVID patients (n = 40)**	
**Co-morbidity**	**% In 40 Long COVID patients**
Hypertension	28%
Hyperlipidaemia	25%
Type 2 Diabetes Mellitus	8%
Rosacea	3%
Thrombosis (previous blood clots)	5%
Cardiovascular disease	8%
Psoriasis	3%
COPD	3%
Cancer	8%
Gout	5%
Hypothyroidism	8%

**Table 2:** Microclot parameters including objects/mL, mean area, and microclot area distribution of 20 controls and 40 Long COVID patients. The non-parametric data are expressed as Median[Q1-Q3].

Microclot Parameter	Control Median[Q1-Q3]	Control Min-Max	Long COVID Median[Q1-Q3]	Long COVID Min-Max	*P*-value
**Objects/mL**	21026[10688–57715]	3673–88644	52934[23634–104281]	2226-2686282	*p < 0.05
**Mean area**	185[153–232]	125–289	228[185–316]	84–415	*p < 0.05
**Microclot area distribution**					
**Count within area range(μm**^**2**^**)**					
**0**–**100**	31[16–114]	6–626	100[13–364]	0–5041	**ns**
**100**–**400**	217[114–396]	34–994	460[223–814]	17–25988	*p < 0.05
**400**–**900**	16[11–43]	1–83	48[19–123]	6–7808	**p < 0.01
**900**–**1600**	2[1–4]	0–10	5[1–25]	0–829	*p < 0.05

#### 2.2.4 Data analysis

All flow cytometry data analysis was performed using the IDEAS 6.2 software developed for the Amnis® FlowSight® Imaging Flow Cytometer. For analysis, only ThT positive events were collected during acquisition. A gate was also established to eliminate events with weak fluorescence signal **(see supplementary material)**. For analysis, we included the following microclot parameters: objects/mL, mean area, and area distribution. GraphPad Prism software (version 9.5.0) was used to perform statistical analysis on all data. To determine whether data were normally distributed, the Shapiro-Wilk normality test was applied. Parametric data were analyzed using unpaired, one-tailed t-tests with Welch's correction to test for statistical significance. For non-parametric data, an unpaired, one-tailed Mann-Whitney test was utilized to test for statistical significance. Statistical significance was taken to be at p < 0.05 (and was also marked with asterisks with * = p < 0.05; ** = p < 0.01). Data are represented as either mean ± standard deviation (parametric data) or as median ± [Q1-Q3] (non-parametric data).

## 3 Results

Table 1 shows the sample demographics of the 60 samples included in this cohort, including co-morbidity distributions of the 40 Long COVID samples. The self-reported symptoms are also listed. Three years after the pandemic started, the clinical characterization of Long COVID remains challenging for both clinicians and patients. Apart from the need for clearer definitions due to symptom variability over time, there is currently no clinical pathology test available to identify patients [30,31]. The current Long COVID patients reported significant comorbidities before their acute infection. We recognise that understanding the correlation between co-morbidities and amyloid fibrin(ogen) microclot detection is essential to identify potential risk factors for Long COVID and to establish whether microclots (and clotting pathology in general) are linked to specific underlying health conditions. We acknowledge the necessity to, in the future, also differentiate between clotting pathology due to existing comorbidities and that due Long COVID, as it is crucial to distinguish the unique aspects and potential interactions of the two categories.

Whilst vaccination status is available as noted in Table 1, the specific impact of vaccination on Long COVID microclot formation remains to be further studied. The study's focus on the presence of microclots is therefore independent of vaccination effects. There is a need for further research to identify the potential impact of vaccination on the observed findings.

As for the controls, healthy individuals without known comorbidities or inflammatory diseases were selected. This approach allowed for a direct comparison between the Long COVID patients and healthy controls, providing insights into potential differences in microclot prevalence between the two groups.

While the current study did not directly explore the potential correlation between microclot detection by imaging flow cytometry on one hand and specific symptoms and comorbidities on the other, the findings lay the groundwork for future research to unravel the relationship between different parameters and distinct symptoms. Further research in this direction has the potential to deepen our understanding of the pathophysiology of Long COVID and facilitate the development of improved detection methods.

**Fig. 1:**
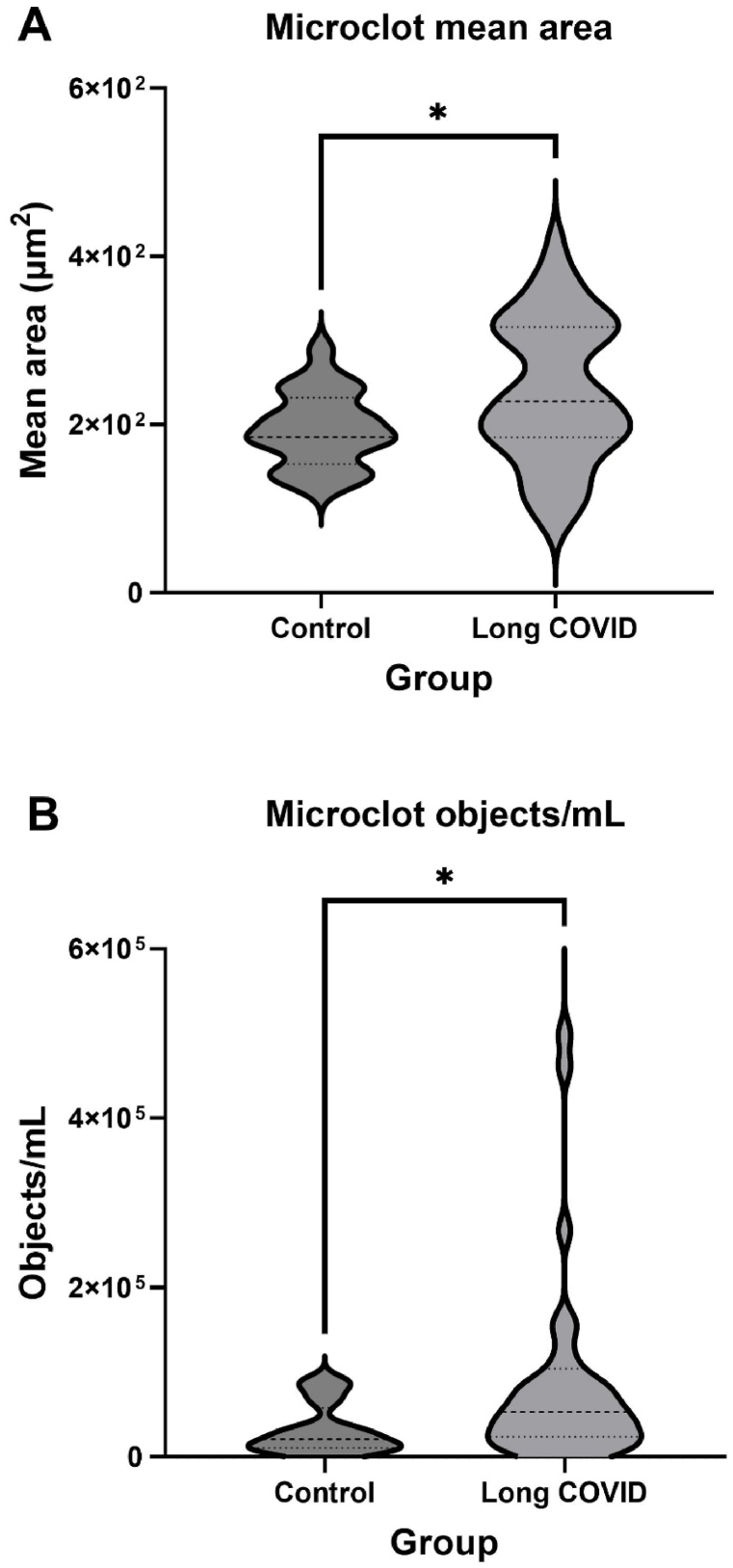
Microclot parameters in controls and Long COVID. **(A)** Microclot mean area - (*p < 0.05), and **(B)** Microclot objects/mL - (*p < 0.05). Graphs are presented as Median[Q1-Q3].

**Fig. 2:**
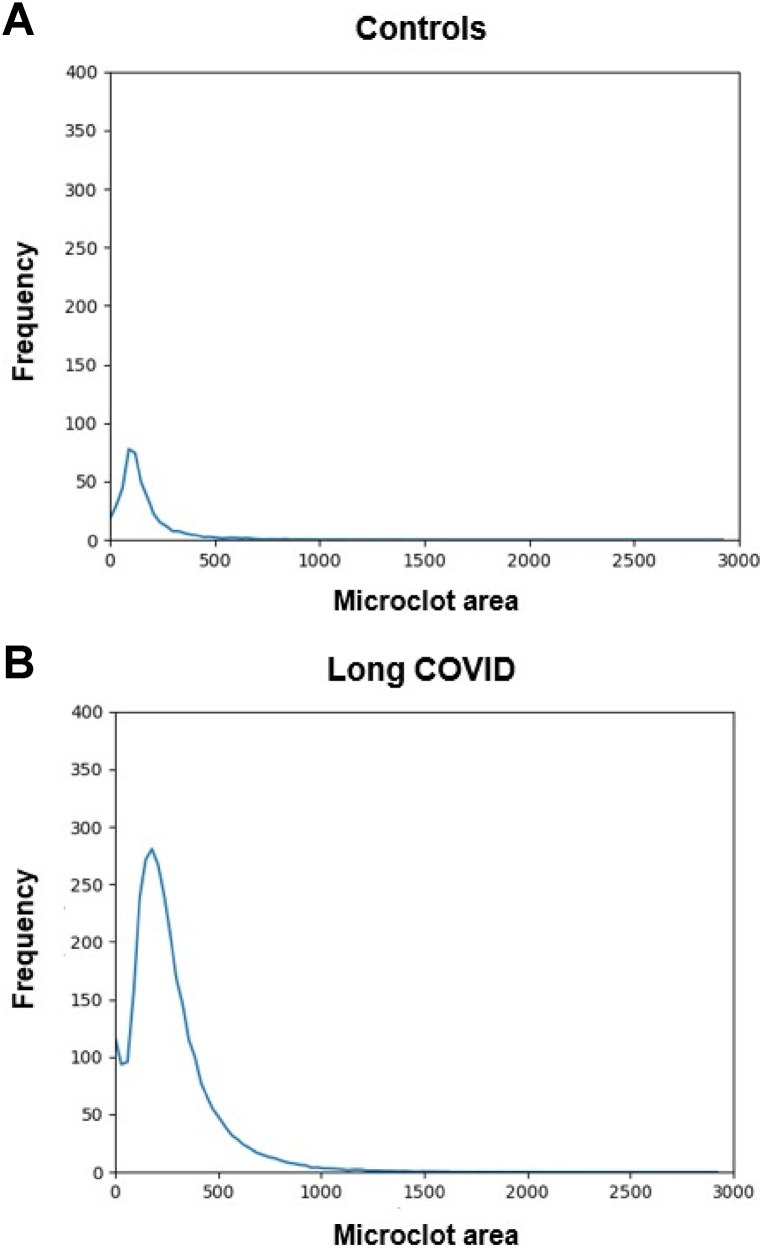
This graph displays the average microclot area distribution of **(A)** control samples (n = 20) and **(B)** Long COVID samples (n = 40). It was created by dividing the brightfield microclot area range into 1000 bins and calculating the average clot-count/frequency per bin for each group of samples. The x-axis indicates the microclot area in the brightfield channel (channel 1), while the y-axis represents the frequency or number of microclots. There is a noticeable difference in the area distribution between the controls and Long COVID patients. The Long COVID patients not only have a greater amount of microclots, but the microclots have a much larger area range than the controls.

Table 2 shows the microclot parameters of interest that were captured, which included: microclot objects/per mL, microclot mean area, and microclot area distribution. As shown in Fig. 1, our results showed that the Long COVID patients had significantly higher concentration of microclots (*p < 0.05) as well as a higher microclot mean area (*p < 0.05) compared to controls. This suggests that the number of microclots, and their size are important parameters to consider. Furthermore, we considered microclot area distribution to be an important parameter as it takes microclot count and size into consideration. Fig. 2 displays and compares the microclot area distribution between controls and Long COVID patients. Here a clear dissimilarity is observed in the microclot area distribution between the control and Long COVID patients. The Long COVID patients exhibit a much larger frequency (number of microclots) and have a broader area range in comparison to the control group, especially in the larger area range.

To quantify the differences seen in the microclot area distribution, we determined the number of microclots falling within each area range **(**Table 2**)**. These area ranges are not based on any existing reference ranges but are merely a practical way of quantifying where the significant differences lie in the area distribution. The first area range was between 0 and 100 μm^2^ (10 μm), which is the approximate equivalent to a capillary diameter. Thereafter, each area range increased by 10 μm. As seen in Table 2, the Long COVID patients showed a significant increase in all, but one area range compared to controls. This provided interesting insights as we realized that whilst bigger microclots are obviously problematic due to the potential for vessel blockage, an increase in smaller microclots has potential clinical significance as well. These smaller microclots may not cause a significant blood vessel blockage but may contribute to platelet activation and endothelial damage.

**Fig. 3:**
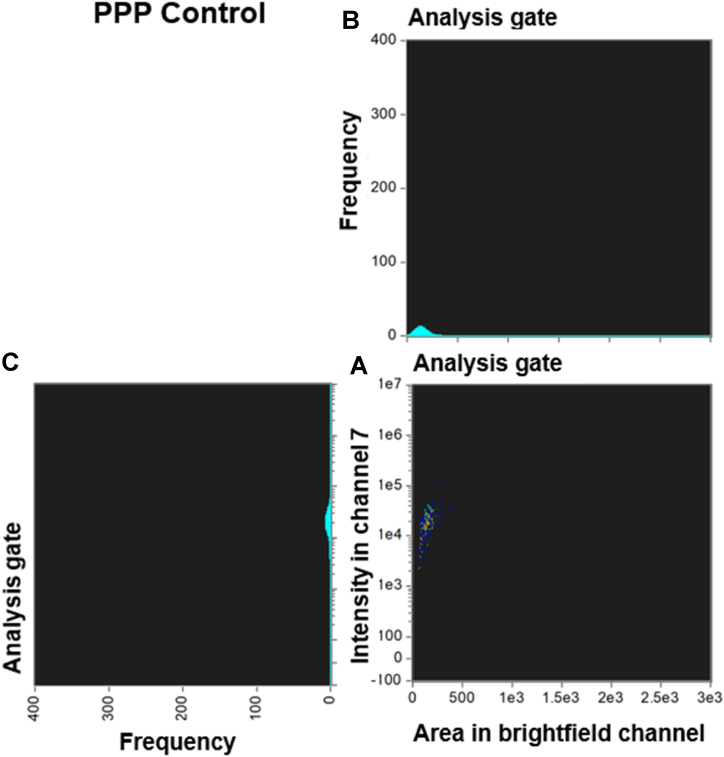
Density dot plot and corresponding cytograms of a representative control using the analysis template. **(A)** The density dot plot (bottom right) depicts microclot events, as defined by the intensity mask. The x-axis of the density dot plot represents the area of intensity in channel 7, while the y-axis represents the intensity in channel 7. **(B)** The top right graph displays a cytogram projection of the x-axis on the density dot plot, **(C)** while the bottom left graph represents a cytogram projection of the y-axis on the density dot plot. Both cytograms depict the frequency or number of events along each axis. As demonstrated in the density dot plot, the microclot events within the control sample are very low, resulting in a very low frequency in both cytograms. The number of objects per milliliter is 28329 objects/mL. **Abbreviations:** PPP, platelet poor plasma.

**Fig. 4:**
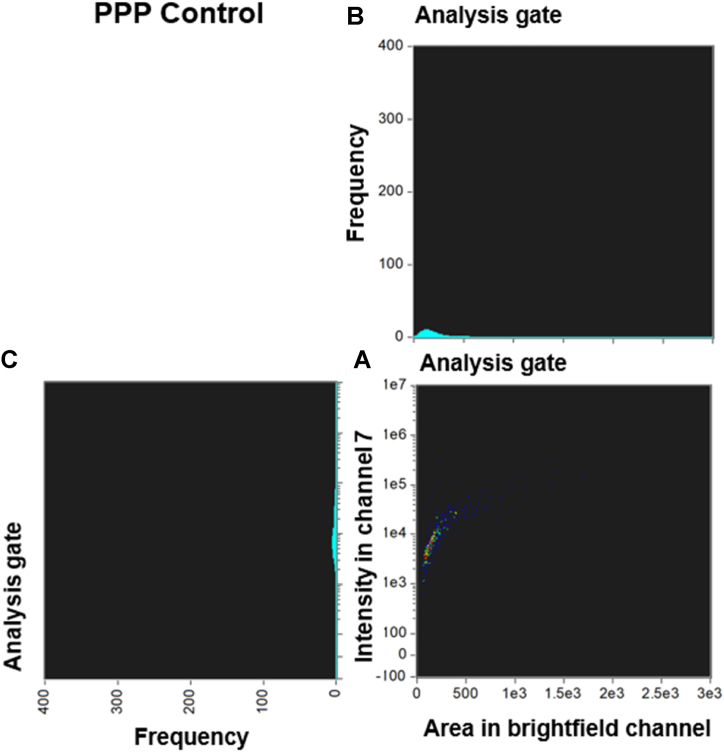
Density dot plot and corresponding cytograms of a representative control using the analysis template. **(A)** The density dot plot (bottom right) depicts microclot events, as defined by the intensity mask. The x-axis of the density dot plot represents the area of intensity in channel 7, while the y-axis represents the intensity in channel 7. **(B)** The top right graph displays a cytogram projection of the x-axis on the density dot plot, **(C)** while the bottom left graph represents a cytogram projection of the y-axis on the density dot plot. Both cytograms depict the frequency or number of events along each axis. As demonstrated in the density dot plot, the microclot events within the control sample are relatively low, resulting in a relatively low frequency in both cytograms. The number of objects per milliliter is 30888 objects/mL. **Abbreviations:** PPP, platelet poor plasma.

**Fig. 5:**
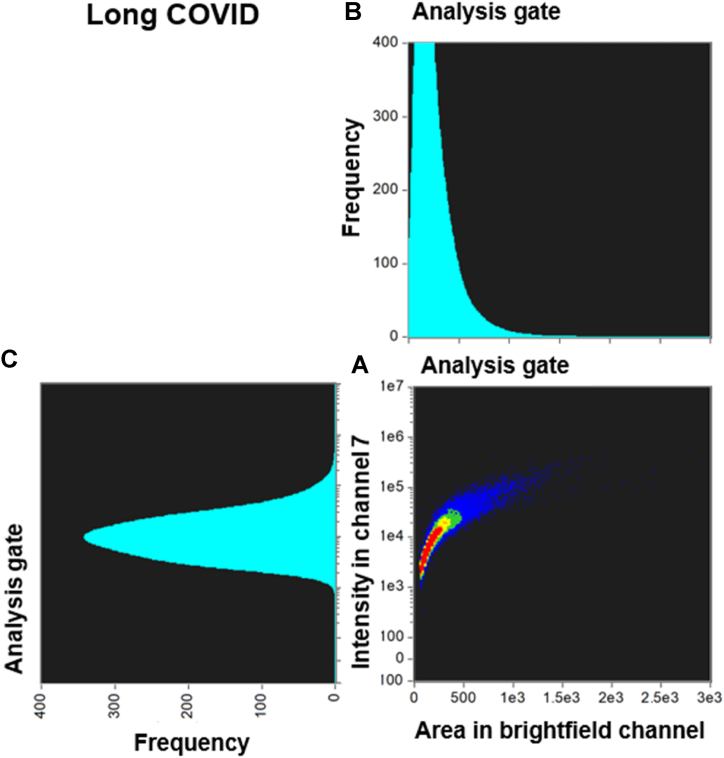
Density dot plot and corresponding cytograms of a representative Long COVID using the analysis template. **(A)** The density dot plot (bottom right) depicts microclot events, as defined by the intensity mask. The x-axis of the density dot plot represents the area of intensity in channel 7, while the y-axis represents the intensity in channel 7. **(B)** The top right graph displays a cytogram projection of the x-axis on the density dot plot, **(C)** while the bottom left graph represents a cytogram projection of the y-axis on the density dot plot. Both cytograms depict the frequency or number of events along each axis. As demonstrated in the density dot plot, the microclot events within the Long COVID sample are high, resulting in a high frequency in both cytograms. The number of objects per milliliter is 2547977 objects/mL.

**Fig. 6:**
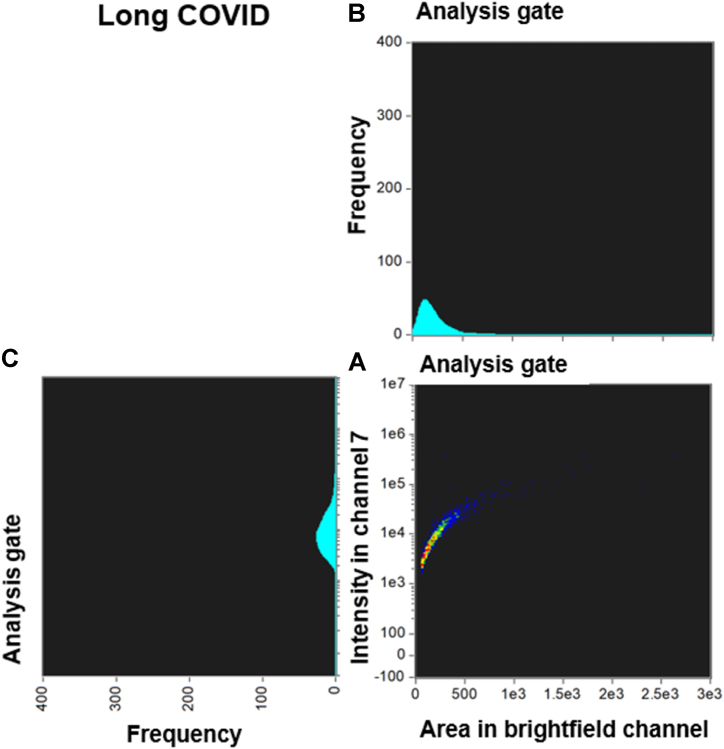
Density dot plot and corresponding cytograms of a representative Long COVID using the analysis template. **(A)** The density dot plot (bottom right) depicts microclot events, as defined by the intensity mask. The x-axis of the density dot plot represents the area of intensity in channel 7, while the y-axis represents the intensity in channel 7. **(B)** The top right graph displays a cytogram projection of the x-axis on the density dot plot, **(C)** while the bottom left graph represents a cytogram projection of the y-axis on the density dot plot. Both cytograms depict the frequency or number of events along each axis. As demonstrated in the density dot plot, the microclot events within the Long COVID sample are very high, resulting in a very high frequency in both cytograms. The number of objects per milliliter is 160054 objects/mL.

Fig. 3, Fig. 4 and Fig. 5, Fig. 6 compare the density dot plots and corresponding cytograms of representative controls and Long COVID patients utilizing the analysis template in IDEAS. The density dot plot and cytograms display all microclot events within the ThT positive gate post-removal of events with weak fluorescence signal **(see supplementary material)**. The density dot plots located in the bottom right-hand corner of Fig. 3, Fig. 4, Fig. 5, Fig. 6 demonstrate the correlation between the area of intensity in channel 7 (x-axis) and the fluorescence intensity in channel 7 (y-axis). The top right graphs portray a cytogram projection of the x-axis on the density dot plot, while the bottom left graphs display a cytogram projection of the y-axis on the density dot plot. As illustrated in Fig. 3, Fig. 4, the density dot plot of two controls indicates a relatively low number of events and is primarily situated towards the lower end of the x and y axis. Consequently, the corresponding cytograms also exhibit a relatively low frequency across both axes. Fig. 5, Fig. 6 illustrate that, in contrast to the controls, the Long COVID samples display a higher number of events in the density dot plots and tend to extend towards the upper end of the x and y axes, signifying an increase in both microclot area and fluorescence intensity. Equivalently, the corresponding cytograms in Fig. 5, Fig. 6 also demonstrate an increased frequency across both axes.

**Fig. 7:**
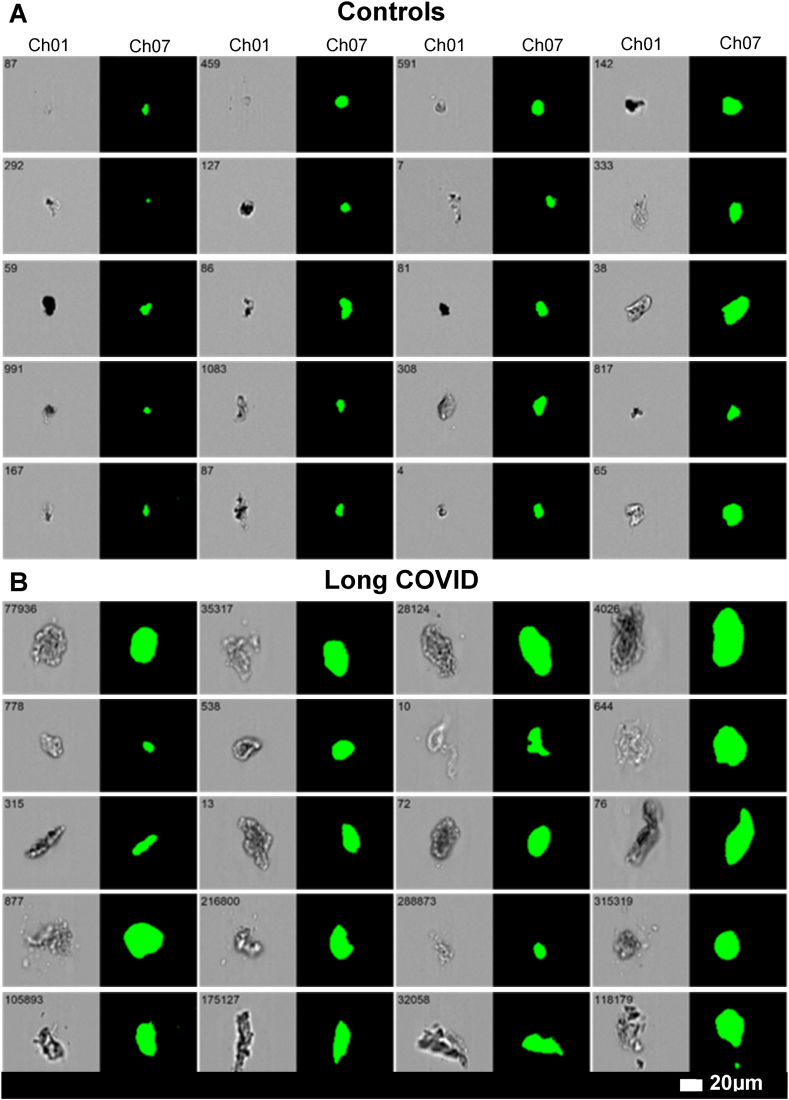
Representative micrographs of microclots in **(A)** controls and **(B)** Long COVID patients using the imaging flow cytometer. The brightfield images are displayed in Channel 1(Ch01), and fluorescence intensity due to ThT binding in Channel 7(Ch07). All images were captured using a 20× objective. The event number is displayed in the top left corner of each image.

Lastly, Fig. 7 shows representative images of microclots from both controls and Long COVID samples. Since we used an imaging flow cytometer in this study, we were able to visualize each microclot in both the brightfield channel (Channel 01) as well as the fluorescence channel (Channel 07). From the images in Fig. 7, it is evident that controls exhibit smaller microclots with a lower fluorescence intensity. Conversely, Long COVID patients tend to have larger microclots that are amyloid in nature, and therefore also have a higher fluorescence intensity.

## 4 Discussion

This study represents a preliminary investigation into the detection of fibrin(ogen) amyloid microclots using imaging flow cytometry in Long COVID patients. Previously, we have used fluorescence microscopy and a semi-quantitative grading system to evaluate the presence of significant microclotting in Long COVID compared to healthy individuals [[2], [3], [4],^6^,^26^]. This method provided good insight into the morphology and size of the microclots but lacked statistical robustness, objectiveness, and throughput. Therefore, we decided to use an imaging flow cytometer that allowed us to combine cell imaging as well as the high-event-rate nature of a conventional flow cytometer. Flow cytometry is a widely used analytical approach for counting, analysing, and segregating cells that are suspended in a fluid stream [28,32,33]. Due to its quantitative and multiparametric characteristics, as well as the analytical capacity to process up to 50,000 cells per second, flow cytometry is widely recognized as the gold standard technique for counting and characterizing cells in complex samples [32]. At its most fundamental level, flow cytometry entails the sequential measurement of individual cells or microscopic particles as they as they move through an optical probe volume at a high-speed rate [32].

In contrast to the purely numerical quantitative measurements provided by conventional flow cytometry, analysing cell images can also help eliminate erroneous results, such as distinguishing between cells and debris [32,34,35]. This approach can lead to more accurate gating and results [32,34]. Furthermore, conventional flow cytometers are incapable of providing spatially resolved information, which is frequently vital for quantifying intricate cellular phenotypes [32,35,36]. Imaging flow cytometry also allows individual cells to be captured in multiple fluorescence channels, as well as brightfield (transmitted light) and darkfield (scattered light) channels [35,36].

In the current study, we chose to focus on those parameters that we considered best to characterize the extent of the microclots. We included objects/mL, mean area, and area distribution as representative parameters for this analysis. We have previously suggested that clotting pathology is mainly caused by larger microclots that trap inflammatory molecules [4]. Therefore, in addition to microclot concentration, the mean area of the microclots as well as the area distribution is important to consider.

As illustrated in Table 2 and Fig. 1, our flow cytometry analysis confirmed that amyloid microclot objects/mL (*p < 0.05) and microclot mean area (*p < 0.05) were significantly increased in Long COVID compared to controls. Furthermore, Table 2 and Fig. 2 indicate that there are significant differences in the microclot area distribution between controls and Long COVID. Hence in Long COVID not only is the number and concentration of microclots significantly higher, but their size is significantly larger when compared to controls.

The graphs depicted in Fig. 3, Fig. 4, Fig. 5, Fig. 6 provide additional evidence of significant variations in the density and distribution of microclots between the control group and individuals with Long COVID. Specifically, the analyses demonstrate that Long COVID patients have a greater number of events occurring within the density dot plots and a higher incidence of events falling to the right side of the graph, which indicates larger sized amyloid microclots. Examining the acquisition graphs, we also observed a significant amount of background events in Long COVID patients in comparison to controls. By looking at the brightfield images, we determined that some of this background activity could be attributed to cellular debris from damaged endothelial cells and fibrinogen strands. These findings align with our previous publications, which suggest the presence of endothelial damage in Long COVID patients [3,4,7,26].

In Fig. 7, micrographs of microclots in controls and Long COVID patients are compared using the imaging flow cytometer. The results demonstrate that even at a 20× objective, there are notable variations in size and fluorescence between Long COVID and controls.

Flow cytometry has been utilized in pathology laboratories for a considerable amount of time, making it a more appropriate and feasible means of identifying microclots in people with Long COVID and other conditions with clotting pathologies. Here we have demonstrated significant differences in the concentration, mean area, and count in various area ranges between Long COVID and controls. These variables have the potential to guide clinical decisions about diagnosis and treatment. The imaging cytometer measurements use both size and fluorescence. Thus, since forward scatter depends mainly on the size of scattering objects (as well as their refractive index), we can anticipate the ability of non-imaging flow cytometers to prove effective in making similar disseminations for the purposes of high-throughput diagnostics.

This study shows the potential of imaging flow cytometry as a method for detecting microclots. Our findings also indicate that microclot parameters such as objects/mL, mean area, and area distribution, may be useful in identifying clotting abnormalities in Long COVID patients. By measuring these variables, clinicians can potentially guide treatment decisions by tailoring therapies to address the specific clotting abnormalities observed in a patient. In addition, these parameters may be useful in monitoring the effectiveness of treatment by tracking the changes in them. It is important to emphasize that further research consisting of analysis of large numbers of control and disease samples is necessary to establish standardized reference ranges and to determine the reliability of this method as a diagnostic tool.

Overall, our novel, rapid, cell-free flow cytometric detection method, utilizing imaging flow cytometry, reveals the presence of amyloid fibrin(ogen) microclots in platelet poor plasma. These microclots represent a potential breakthrough in clinical diagnostics, offering valuable insights into clotting pathologies. With its promising applicability, this method holds the potential to become a diagnostic tool for individuals with Long COVID, enabling comprehensive evaluation of microclot presence, concentration, and size.

### 4.1 Limitations of the study

This study serves as an initial exploration into the potential of imaging flow cytometry as a method for detecting microclots, particularly amyloid fibrin(ogen) microclots in Long COVID patients. Whilst the findings provide promising insights, it is important to acknowledge that the presence of large amyloid fibrin(ogen) microclots in Long COVID patients has been previously demonstrated using fluorescence microscopy. The current study highlights the advantages of employing a more accessible, quantitative, and high-throughput detection method for these microclots.

To fully establish the clinical significance and wider applicability of this detection method, further research is needed. Investigations should be conducted using larger and more diverse datasets from individuals with various inflammatory conditions and comorbidities, allowing for a more comprehensive assessment of the method's performance. This would help determine whether the detection method exhibits a consistent bimodal distribution between healthy and disease states and the extent to which features of the microclot distributions can be said to be disease specific. It will also be crucial to explore any potential differences in results between fresh and stored samples. Understanding the impact of sample storage on the detection method's outcomes will help validate its reliability and reproducibility in practical clinical settings.

Overall, these limitations underscore the need for future studies with enhanced sample sizes, diverse patient groups, and the investigation of sample storage effects to elucidate the full potential and clinical utility of imaging flow cytometry in detecting amyloid fibrin(ogen) microclots in various inflammatory conditions, including Long COVID.

## Consent for publication

All authors approved submission of the paper.

## Author contribution statement

Simone Turner: Conceived and designed the experiments; Performed the experiments; Analyzed and interpreted the data; Wrote the paper.

Gert J Laubscher; M Asad Khan: Analyzed and interpreted the data; Wrote the paper.

Douglas B Kell: Analyzed and interpreted the data; Contributed reagents, materials, analysis tools or data; Wrote the paper.

Etheresia Pretorius: Conceived and designed the experiments; Analyzed and interpreted the data; Contributed reagents, materials, analysis tools or data; Wrote the paper.

## Data availability statement

Data will be made available on request.

## Declaration of competing interest

The authors declare the following financial interests/personal relationships which may be considered as potential competing interests:Competing interests

EP is a director of Biocode Technologies; ST is the COO at Biocode Technologies. The other authors have no competing interests to declare.
