# Supplementary material for: Accelerating discovery: A novel flow cytometric method for detecting fibrin(ogen) amyloid microclots using long COVID as a model
Source: Heliyon. 2023 Aug 29;9(9):e19605. doi: 10.1016/j.heliyon.2023.e19605 (PMC10558872; doi:10.1016/j.heliyon.2023.e19605)
Supplement: Multimedia component 1 [file mmc1.docx]

**Rapid flow cytometric detection of fibrin amyloid microclots in Long COVID**

**Supplementary material**

This supplementary document provides detailed information about the gating strategy and masking used in the study. **Figure 1-3** shows the gating strategy for the acquisition template, whereas **Figure 4-5** illustrates the gating strategy for the analysis. **Figure 6** shows the masking technique used for this study.

**1.Gating strategy for the acquisition template**

**Figure 1** shows the creation of a Thioflavin (ThT) positive gate using a negative control using double distilled water and the fluorescent marker, ThT. The expectation is that there will be little background noise and no events within the ThT positive gate since it is a negative control. To validate the gating strategy, a standard platelet poor plasma (PPP) sample without ThT was used **(Figure 2)**. This sample was expected to have more background noise due to it being PPP and not water, but the ThT gate was still able to exclude most background events. Finally, a standard PPP with ThT was used **(Figure 3)**, where multiple events were picked up in the ThT channel due to ThT binding to amyloid microclots. Overall, our gating strategy appears to have been successful, with the acquisition gate effectively excluding background events and picking up ThT binding to amyloid microclots.

In **Figure 1-3**, the density dot plots in the bottom right corner of each figure show the relationship between the area of the event in the brightfield channel (x-axis) and the fluorescent intensity of the event in channel 2 (y-axis). The top right graphs show a cytogram projection of the x-axis on the density dot plot, while the bottom left graphs show a cytogram projection of the y-axis on the density dot plot. The cytogram projections only illustrate the events within the ThT+ gate. These graphs are important for visualizing the data and confirming that the gating strategy is working as intended. By looking at the density dot plots, it is possible to identify the events that fall within the desired gating region, which in this case is the ThT+ gate. The cytogram projections provide a summary of the data distribution along each axis.

**Figure 1:** The acquisition template showing the ThT+ gate that was generated using the negative control (double distilled water and ThT) as a reference, illustrated as density dot plot (bottom right) and cytograms of density dot plots (top right and bottom left). The density dot plot shows all the events, whereas the cytograms only illustrate the events within the ThT+ gate. The x-axis represents the area in the brightfield channel, and the y-axis represents the intensity in channel 2. The frequency refers to the number of events. The ThT+ gate effectively excluded background events and did not register any noteworthy occurrences in the negative control, which demonstrates that the acquisition gate was properly established. The objects per milliliter in total is 73318 objects/mL and for the ThT+ gate, 151 objects/mL. **Abbreviations**: ThT, thioflavin T.


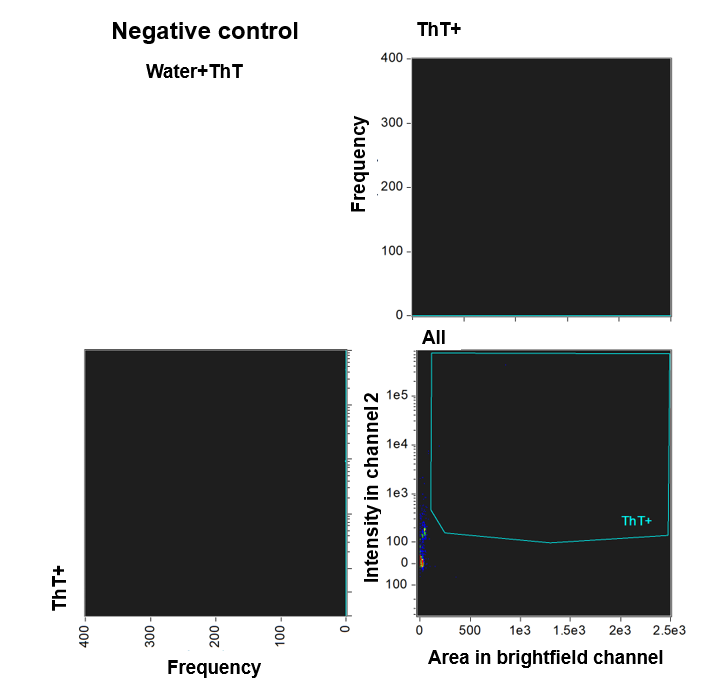


**Figure 2:** The acquisition template showing the ThT+ gate that was generated using the negative control (PPP without ThT) as a reference, illustrated as density dot plot and cytograms of density dot plots. The density dot plot (bottom right) shows all the events, whereas the cytograms (top right and bottom left) only illustrate the events within the ThT+ gate. The x-axis represents the area in the brightfield channel, and the y-axis represents the intensity in channel 2. The frequency refers to the number of events. The acquisition gate successfully eliminated most events, thus confirming the appropriate establishment of the acquisition gate. The objects per milliliter in total is 6223118 objects/mL and for the ThT+ gate, 6293 objects/mL. **Abbreviations**: ThT, thioflavin T.

**
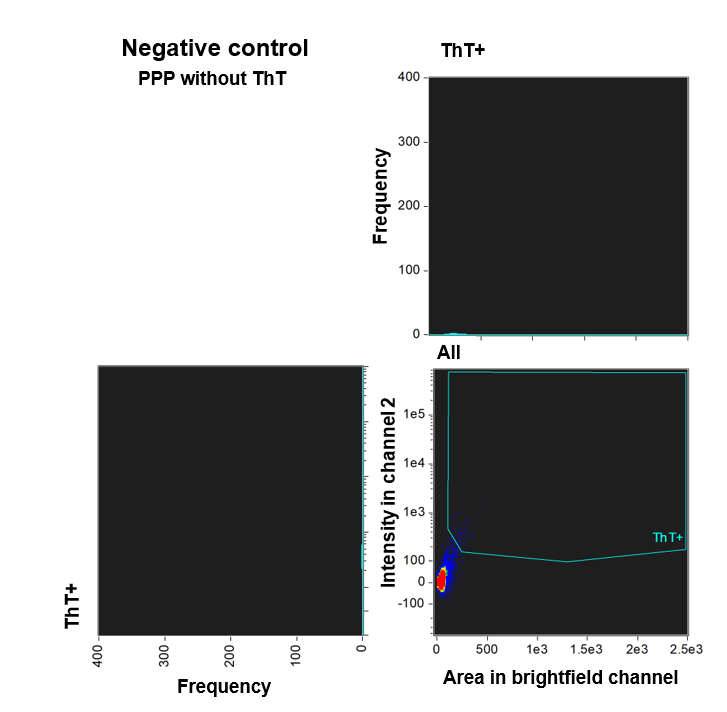
**

**Figure 3:** The acquisition template showing the ThT+ gate that was generated using a standard PPP control with ThT, illustrated as density dot plot (bottom right) and cytograms of density dot plots (top right and bottom left). The density dot plot shows all the events, whereas the cytograms only illustrate the events within the ThT+ gate. The x-axis represents the area in the brightfield channel, and the y-axis represents the intensity in channel 2. The frequency refers to the number of events. As anticipated, the ThT+ gate recorded several incidents due to the binding of ThT to amyloid microclots. Consequently, the ThT+ gate comprises all the fluorescent signal in channel 2 (excitation:405nm; emission:435-505), and further refinement was necessary during the analysis to accurately identify microclot events. The objects per milliliter in total is 12022589 objects/mL and for the ThT+ gate, 75401 objects/mL. **Abbreviations**: PPP, Platelet poor plasma; ThT, thioflavin T.


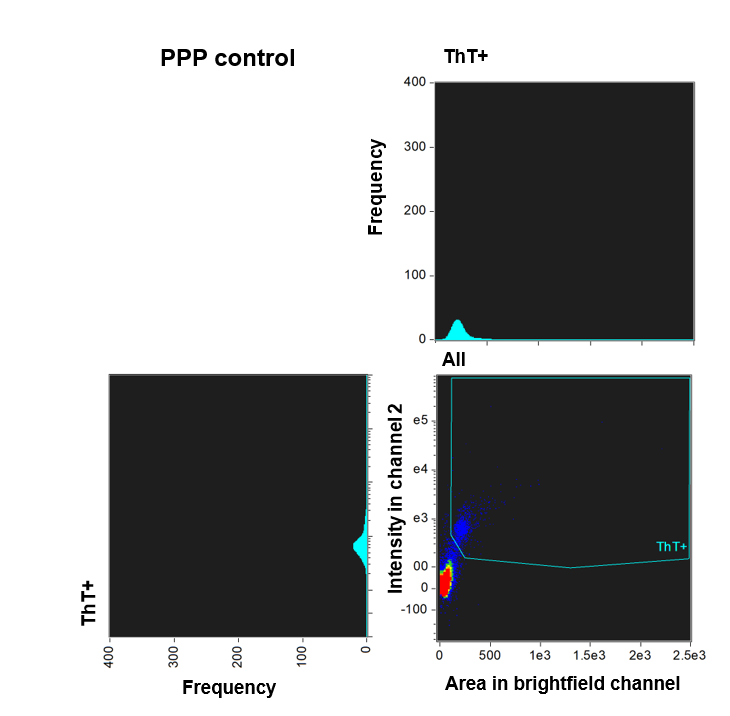


**2.Gating strategy for the analysis template**

During the acquisition process, events that were positive for ThT were collected for analysis. However, it was observed that some of these events exhibited a weak fluorescent signal, potentially leading to less accurate results. To mitigate this issue, a second gate was introduced during the analysis phase. This gate specifically focuses on events with a robust fluorescent signal in channel 7, ensuring that only events with strong fluorescence are considered. By implementing this second gate, the analysis is refined, leading to the inclusion of only those events that truly possess amyloid characteristics, thereby enhancing the reliability of the findings.

**Figure 4:** Analysis template, which excludes weak fluorescent signals. **(A)** Depicts a dot plot showcasing the analysis gate. **(B)** Displays a density dot plot after applying the analysis gate, effectively excluding events with low fluorescent signal. **Abbreviations**: ThT, thioflavin T.

**
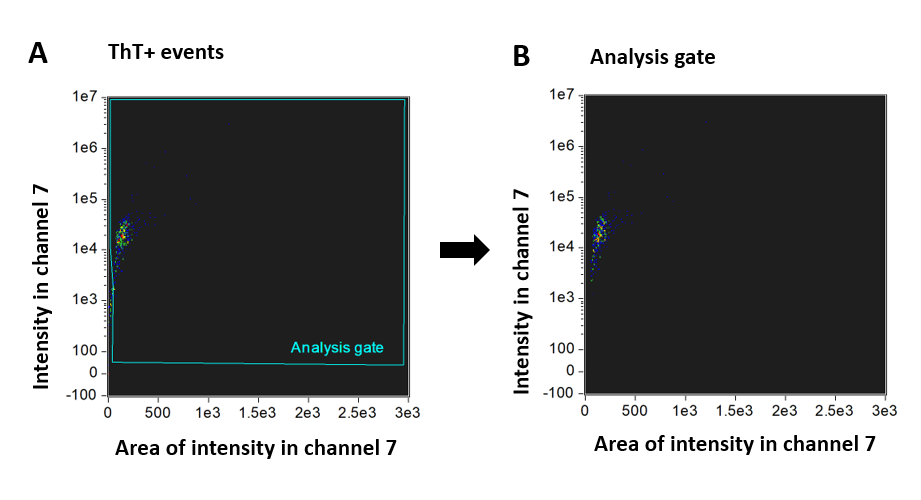
**

**Figure 5:** Density dot plot and corresponding cytograms of a PPP control using the analysis template. The analysis template excluded events that had a weak fluorescent signal in channel 7. The density dot plot (bottom right) depicts microclot events, as defined by the intensity mask. The x-axis of the density dot plot represents the area of intensity in channel 7, while the y-axis represents the intensity in channel 7. The top right graph displays a cytogram projection of the x-axis on the density dot plot, while the bottom left graph represents a cytogram projection of the y-axis on the density dot plot. Both cytograms depict the frequency or number of events along each axis. As demonstrated in the density dot plot, the microclot events within the PPP control sample are low, resulting in a low frequency in both cytograms. The objects per milliliter after excluding events with weak fluorescent signal is 28329 objects/mL. **Abbreviations:** PPP, platelet poor plasma.


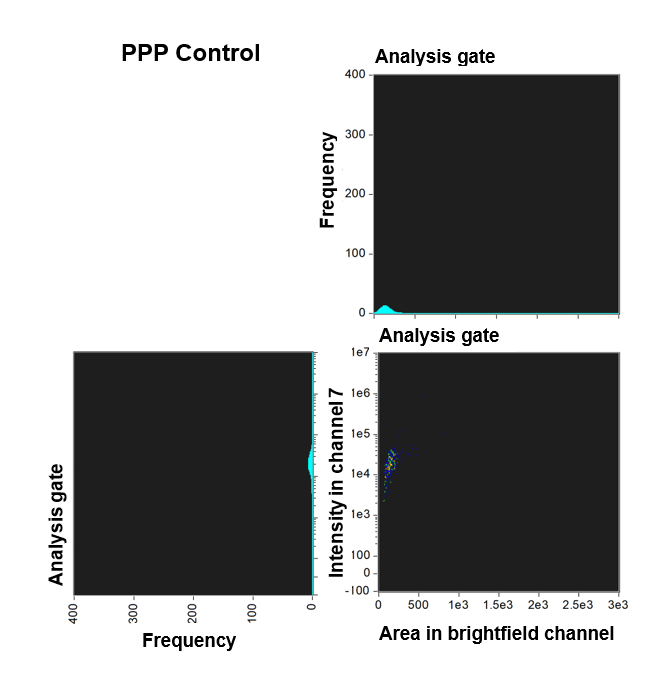


**3. Masking strategy for analysis**

As shown in **Figure 6**, two masks were created for analysis: the brightfield mask for channel 1, and an intensity mask for channel 7. The intensity mask distinguished intensity values above a specific threshold in channel 7, as well as a feature that evaluated the intensity of channel 7 within the mask to eliminate weakly-stained events and background noise.

**Figure 6:** **(A)** Unmasked microclots in brightfield channel (channel 1) and ThT fluorescent channel (channel 7). **(B)** Masked microclots (blue overlay) using the brightfield mask in channel 1 and intensity mask in channel 7. **Abbreviations:** ThT, Thioflavin T.

**Masked**

**Unmasked**

Channel 7

Channel 1


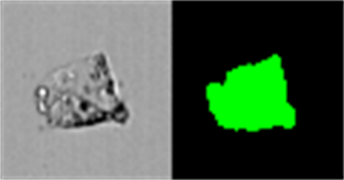

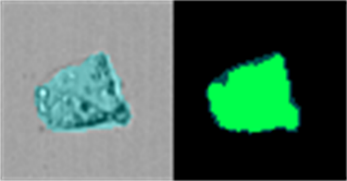

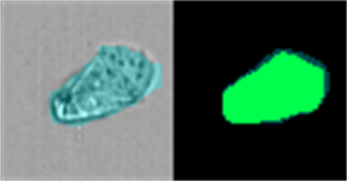

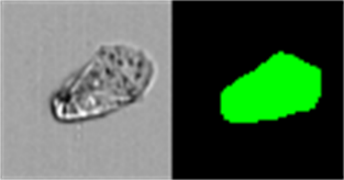

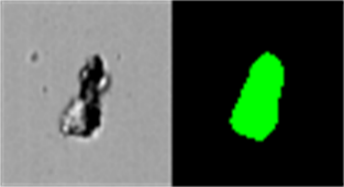

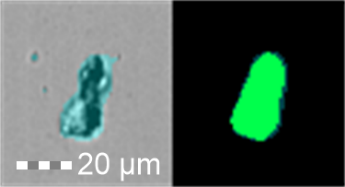


**20µm**

**A**

**B**
